# A preliminary investigation into the immune cell landscape of schistosome‐associated liver fibrosis in humans

**DOI:** 10.1111/imcb.12490

**Published:** 2021-08-06

**Authors:** Yu Zhang, Junhui Li, Hao Li, Zhaoqin Zhou, Chen Guo, Jie Jiang, Yingzi Ming

**Affiliations:** ^1^ Transplantation Center The Third Xiangya Hospital Central South University Changsha Hunan China; ^2^ Engineering and Technology Research Center for Transplantation Medicine of National Health Commission Changsha Hunan China

**Keywords:** liver fibrosis, *Schistosomiasis japonicum*, single‐cell sequencing

## Abstract

Schistosomiasis is a widespread helminth disease that poses a heavy social and economic burden on people worldwide. Advanced schistosomiasis often develops into schistosome‐associated liver fibrosis, the pathogenesis of which remains unclear. This study aimed preliminarily to profile immune cells of schistosome‐associated liver fibrosis using single‐cell RNA sequencing. Three patient groups were enrolled, including an *Schistosomiasis japonicum* (SJ) group (*n* = 1), a chronic liver failure (CLF) group (*n* = 3) and a healthy control (HC) group (*n* = 2), revealing 17 cell clusters out of 20 093 cells. From these limited datasets, it was observed that T cell(1), mononuclear phagocytes‐1 and dendritic cells (DCs) were higher in the SJ group. CAVIN2^+^ MP(2) was the predominant cell type in the MP subset of the SJ group (53%), and was higher than that in both the CLF (8%) and HC (1%) groups. Kupffer cell marker genes (*CD163*, *MARCO* and *TIMD4*) were enriched in caveolae‐associated protein 2 (*CAVIN2*)^+^ MP(2), which was also an important source of *TGFB1*. The KEGG pathways of *CAVIN2* ^+^ MP(2) indicated that they were associated with lysosome, endocytosis, phagosome and antigen processing and presentation. The preliminary study showed that granzyme B (*GZMB*)^+^ T cell(1) and ankyrin repeat domain‐containing protein 36B^+^ T cell(3) were the most common T cells in the SJ group (50% and 32%, respectively). The KEGG pathways of *GZMB* ^+^ T cell(1) were mainly related to natural killer cell‐mediated cytotoxicity. The percentage of ring1 and YY1 binding protein (*RYBP*)^+^ DC(1) was higher in the SJ group (57%) than in the CLF (16%) and HC (6%) groups. The KEGG pathway of *RYBP* ^+^ DC(1) was related to Fc gamma R‐mediated phagocytosis and antigen processing and presentation. Overall, *CAVIN2* ^+^ Kupffer cells were the main source of *TGFB1*, consisting primarily of mononuclear phagocytes in the livers of the SJ group subjects and potentially playing an irreplaceable role in hepatic fibrosis of schistosomiasis.

## Graphical abstract

The data in this study provide a reference map of the liver immune landscape of patients with schistosoma‐associated liver fibrosis.

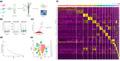

## INTRODUCTION

Liver fibrosis is a major complication of schistosomiasis, the second most common helminth disease in the world.^1^ One type of liver fibrosis, known as schistosoma‐associated liver fibrosis, is induced by granulomatous inflammation around eggs deposited in the liver.^2^, ^3^ *Schistosoma japonicum*, *S*. *mansoni* and *S*. *mekongi* are the main species that will lead to schistosoma‐associated liver fibrosis.^4^ Importantly, extensive liver fibrosis results in portal hypertension, a leading cause of death in patients with schistosomiasis.^5^ However, there is currently no effective treatment for schistosoma‐associated liver fibrosis, highlighting the urgent need to develop novel antifibrotic therapies for patients with schistosomiasis with this condition.

The pathogenesis of schistosoma‐associated liver fibrosis is complex and multifactorial, and emerging studies have demonstrated the involvement of both innate and adaptive immune cells.^6^ Both murine and human schistosomiasis studies have found that macrophages, T cells, B cells, neutrophils, eosinophils, natural killer (NK) cells, innate lymphocytes and other immune cells play a crucial role in schistosoma‐associated liver fibrosis.^7^, ^8^, ^9^, ^10^, ^11^, ^12^, ^13^ However, functional and phenotypic diversity in the immune cells of animal models cannot fully reflect the fact that peripheral blood‐based analysis in humans is incapable of completely revealing liver‐infiltrated immune cells. Thus, the underlying immune mechanism needs to be elucidated. Therefore, furthering our understanding of the immune landscape will not only lead to a better appreciation for the prevention and control of schistosoma‐associated liver fibrosis, but also shed light on treatments for other types of liver fibrosis.

Single‐cell RNA sequencing (scRNA‐seq), a powerful technology capable of profiling the transcriptomes of individual cells, has become widely used in different diseases.^14^, ^15^, ^16^, ^17^ scRNA‐seq can examine cell types and transcriptional patterns that cannot be observed in bulk RNA sequencing.^18^, ^19^ To identify the transcriptomic landscape of immune cells involved in schistosoma‐associated liver fibrosis within the liver, scRNA‐seq analysis was performed on liver samples from patients with cirrhosis with coinfection of *S. japonicum* and hepatitis B virus (HBV), patients with HBV cirrhosis and healthy individuals. We also identified the types and states of the liver‐infiltrating immune cells. Although the sample size was small, bioinformatics analysis revealed that T cells were a major fraction of immune cells within the liver and were upregulated in the SJ group, particularly granzyme B (*GZMB*)^+^ T cells. We also observed a dramatic increase in the caveolae‐associated protein 2 (*CAVIN2*)^+^ macrophage characterized by the high expression of transforming growth factor B1(*TGFB1*) (a master profibrotic gene) in the SJ group.^20^ In addition, we noted that DCs that used ring1 and YY1 binding protein (*RYBP*) as a marker gene were the dominant cluster in the SJ group. Further analysis indicated that distinct signaling pathways, mainly related to NK cell mediated cytotoxicity and antigen processing and presentation, were activated in members of the SJ group. This preliminary study indicated that the liver‐infiltrating immune cells with unique marker gene expression and specific signaling pathway activation may be helpful for our understanding of the mechanism of schistosoma‐associated liver fibrosis.

## RESULTS

### Single‐cell atlas of the human liver

**Figure 1:**
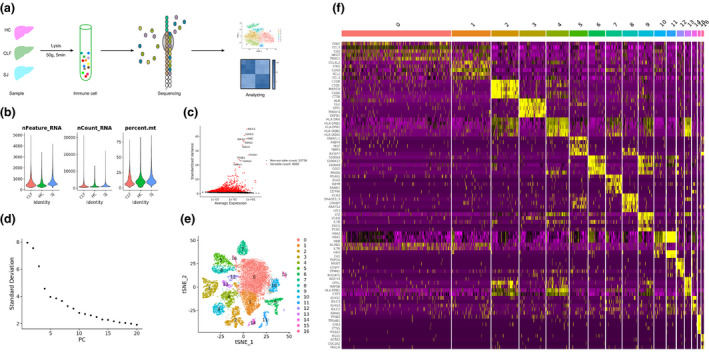
Quality control of single cell from three groups. **(a)** The flowchart of our study including grouping (SJ = 1, CLF = 3, HC = 2), dissociation, sequencing and analyzing. **(b)** Violin plots of different groups about the unique feature counts, the mRNA reads and the percentage of mitochondrial genes. **(c)** Identification of top 4000 variable genes. **(d)** The plot of the first 20 PCs. **(e)** tSNE plots of the 20 093 immune cells for cell clusters. **(f)** Heatmap of top five DEGs of each cluster.

Single cells were isolated from the livers of chronic liver failure (CLF; *n* = 3), *S. japonicum* (SJ; (*n* = 1) and healthy control (HC; *n* = 2) subjects. Following low‐speed centrifugation to remove hepatocytes, the non‐parenchymal cells (NPCs) of the liver were processed for scRNA‐seq, and further biological analysis was performed on the resultant data (Figure 1a). We performed quality control analyses based on the unique feature counts, mRNA reads and the percentages of mitochondrial genes (Figure 1b). We filtered out cells with unique feature counts < 200 or > 10 000, and included cells containing less than 25% of mitochondrial genes. The top 4000 variable genes were selected for principal component analysis. The first 18 Principal Component (PCs) were used to calculate clusters with a resolution of 0.4 for clustering analysis, using the Seurat function “FindClusters” (Figure 1c, d). The tSNE plot finally revealed 17 major cell clusters, and 20 093 cells were included in our study after quality control filters were applied (Figure 1e). The heatmap shows the top five differentially expressed genes of each cluster (DEGs) (Figure 1f).

**Figure 2:**
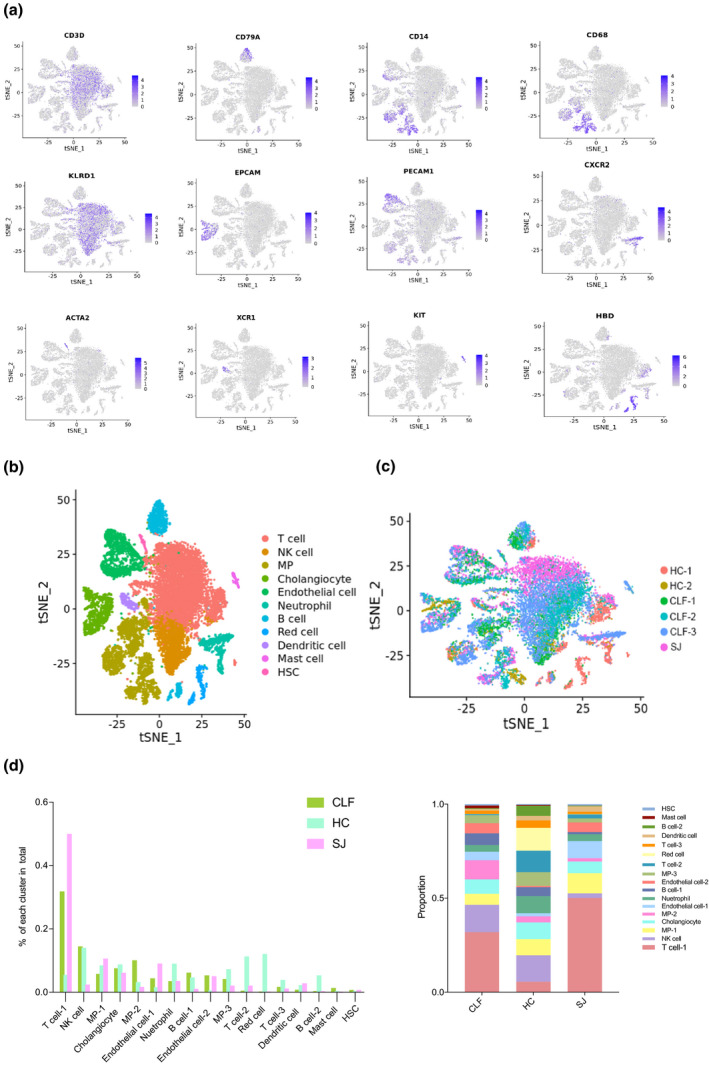
Overview of the 20 093 single cells isolated from liver of three groups (SJ = 1, CLF = 3, HC = 2). **(a)** Expression of cell specific marker genes used to annotate clusters. **(b, c)** tSNE plot of different type cells and three groups. **(d)** The percentage change tendency and contribution of each cell cluster in different groups.

In this preliminary study, we annotated clusters based on the gene expression of cell type‐specific markers (Figure 2a), and identified T cells (3 cell clusters), NK cells (1 cell cluster), mononuclear phagocytes (MP, 3 cell clusters) and cholangiocytes (1 cell cluster), as well as endothelial cells (2 cell clusters), neutrophils (1 cell cluster) and dendritic cells (DCs; 1 cell cluster). We also identified B cells (2 cell clusters), mast cells (1 cell clusters), hepatic stellate cells (HSCs, 1 cell cluster) and red cells (1 cell cluster) (Figure 2b). The distribution of total cells was observed among the different groups using t‐distributed stochastic neighbor embedding (tSNE; Figure 2c). As shown in Figure 2d, the percentage of T cell(1) in the SJ group was dramatically increased compared with that in the CLF and HC groups. Additionally, MP‐1, DC and endothelial cell‐1 percentages were higher in the SJ group than in the CLF and HC groups.

### *CAVIN2*^+^ macrophages facilitate liver fibrosis in *S. japonicum*

**Figure 3:**
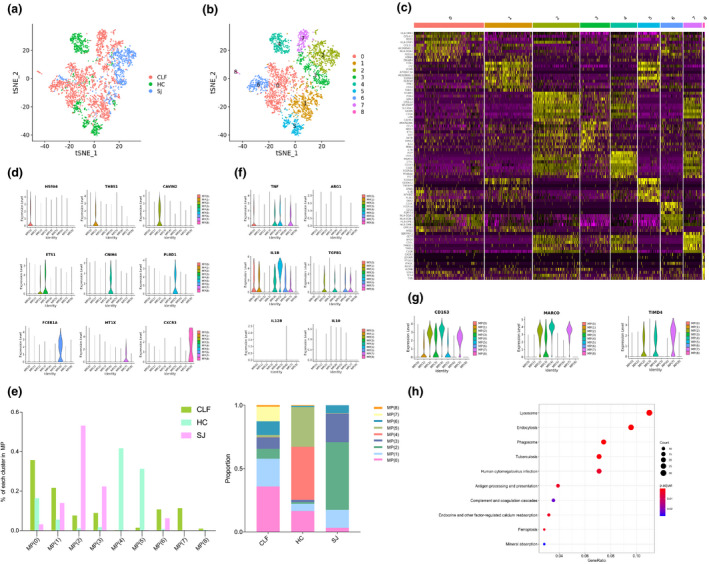
Mononuclear phagocyte clusters. **(a)** tSNE plots of the 3711 MPs for different groups. **(b)** tSNE plots of nine MPs clusters. **(c)** Heatmap of MPs for the top 10 DEGs of each cluster. **(d)** Violin plots of marker genes in each MPs cluster. **(e)** The percentage change tendency and contribution of each MPs cluster in different groups. **(f)** Violin plots of M1 and M2 marker genes in each MPs cluster. **(g)** Violin plots of Kupffer cell marker genes in each MPs cluster. **(h)** Bubble plots of KEGG pathway enrichment data of MP(2).

We detected 3711 MPs and tSNE plots for different types of cirrhosis. All cells were grouped into nine main clusters (Figure 3a, b). The heatmap shows the top 10 DEGs of each cluster (Figure 3c). Marker genes were identified for different clusters, such as *HSPA6* for MP(0), *THBS1* for MP(1), and *CAVIN2* for MP(2), as well as *ETS1* for MP(3), *CNIH4* for MP(4) and *PLBD1* for MP(5). Other marker genes and clusters were *FCER1A* for MP(6), *MT1X* for MP(7) and *CXCR3* for MP(8) (Figure 3d). There were apparent differences in the cluster composition between the groups, with MP(1), MP(2) and MP(3) being the major MPs in the SJ group. The CLF group was mainly composed of MP(0) and MP(1), and the HC group was largely composed of MP(4) and MP(5). Even though the sample size was limited, we observed that there were substantially higher percentages of MP(2) and MP(3) in the SJ group (53% and 22%, respectively) than in the CLF (8% and 9%, respectively) and HC (1% and 2%, respectively) groups (Figure 3e).

Macrophages are classically divided into M1 and M2 macrophages, and different cell clusters have been detected for marker genes of M1 (*TNF*, *IL1B*, *IL12B*) and M2 (*ARG1*, *TGFB1*, *IL10*).^3^, ^21^ *TNF* was expressed in MP(0), MP(4), MP(5) and MP(7). *IL1B* was expressed in all clusters except MP(2), MP(3) and MP(8). *TGFB1* is a fibrosis‐related gene present in MP(2), MP(4), MP(6) and MP(7). *IL12B*, *IL10* and *ARG1* were absent in all cells (Figure 3f). MPs include infiltrating macrophages and resident Kupffer cells in the liver.^22^ MP(2), MP(4) and MP(7) were enriched in the expression of *CD163*, *MARCO* and *TIMD4,* which are marker genes of Kupffer cells (Figure 3g).^23^ We explored the biological processes about the DEGs between MP(2) and other MP subsets. The KEGG pathways were related to lysosome, endocytosis, phagosome and antigen processing and presentation (Figure 3h).

### Increase of *GZMB* ^+^ T cells in the SJ group

**Figure 4:**
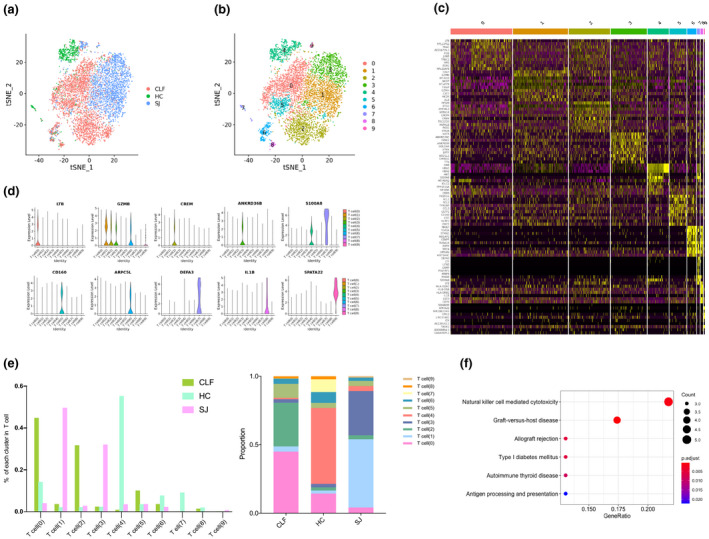
T cell clusters. **(a)** tSNE plots of the 7392 T cells for different groups. **(b)** tSNE plots of 10 T cells clusters. **(c)** Heatmap of T cells for the top 10 DEGs of each cluster. **(d)** Violin plots of marker genes in each T cell cluster. **(e)** The percentage change tendency and contribution of each T cell cluster in different groups. **(f)** Bubble plots of KEGG pathway enrichment data of T cell(1).

We detected 7392 T cells that were grouped into 10 main clusters, with the tSNE plot showing different types of cirrhosis (Figure 4a, b). The heatmap shows the top 10 DEGs of each cluster (Figure 4c). *LTB* was the marker gene for T cell(0), and *GZMB* was mainly expressed in T cell(1), T cell(2), T cell(3) and T cell(6). *S100A8* was present in T cell(4) and T cell(7). *CREM* was the marker gene for T cell(2) in other clusters, just as *ANKRD36B* was for T cell(3) and *CD160* for T cell(5). Other marker genes include *ARPC5L* for T cell(6), *DEFA3* for T cell(7), *IL1B* for T cell(8) and *SPATA22* for T cell(9) (Figure 4d). The SJ group, the patient with coinfection of *S. japonicum* and HBV, mainly consisted of T cell(1) and T cell(3), which were important sources of *GZMB*. However, the HC group was mostly composed of T cell(4) (55%). T cell(0) and T cell(2) were the predominant clusters in the CLF group (45% and 32%, respectively; Figure 4e). KEGG pathway analysis was performed, comparing the DEGs of T cell(1) with those of clusters, as well as those of T cell(3). The T cell(1) pathway was primarily related with NK cell‐mediated cytotoxicity (Figure 4f), and the T cell(3) pathway was involved in spliceosome and lysine degradation (Supplementary figure 1).

### *RYBP*^+^ DC had a strong capacity for antigen presentation in the SJ group

**Figure 5:**
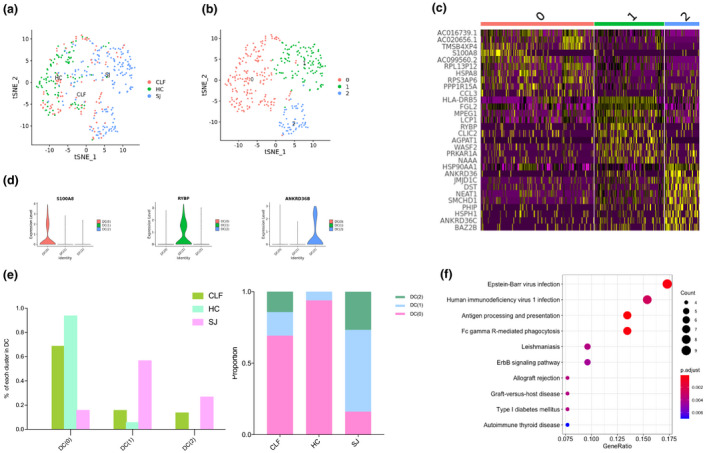
Dendritic cell clusters. **(a)** tSNE plots of the 351 DCs for different groups. **(b)** tSNE plots of three DCs clusters. **(c)** Heatmap of DCs for the top 10 DEGs of each cluster. **(d)** Violin plots of marker genes in each DC cluster. **(e)** The percentage change tendency and contribution of each DC cluster in different groups. **(f)** Bubble plots of KEGG pathway enrichment data of DC(1).

We detected 351 DCs, with three main cell clusters. Different types of cirrhosis were shown in the tSNE plot (Figure 5a, b). The heatmap showed the top 10 DEGs of each cluster (Figure 5c). Upon further searching for marker genes in each cluster, we identified *S100A8* as a specific gene for DC(0), as well as *RYBP* and *ANKRD36* marker genes for DC(1) and DC(2), respectively (Figure 5d). DC(1) formed the dominant cluster in the SJ group (57%) with only one liver sample from cirrhosis patients with coinfection of *S. japonicum* and HBV, while DC(0) formed the main cluster in the CLF (69%) and the HC (94%) groups (Figure 5e). The KEGG pathway analysis of DC(1) revealed significantly enriched pathways related to Fc gamma R‐mediated phagocytosis and antigen processing and presentation (Figure 5f).

### Other immune cells in the SJ group

**Figure 6:**
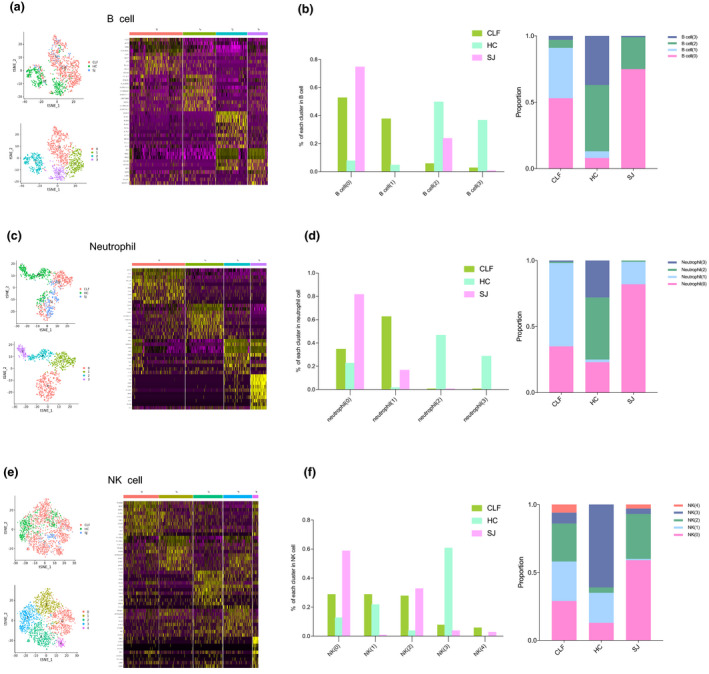
Other immune cell clusters. **(a)** tSNE plots of the 1198 B cells for different groups and four B cell clusters and heatmap of B cell for the top 10 DEGs of each B cell cluster. **(b)** The percentage change tendency and contribution of each B cell cluster in different groups. **(c)** tSNE plots of the 952 neutrophils for different groups and four neutrophil clusters and the heatmap of neutrophils for the top 10 DEGs of each neutrophil cluster. **(d)** The percentage change tendency and contribution of each neutrophil cluster in different groups. **(e)** tSNE plots of the 2231 NK cells for different groups and five NK cell clusters and heatmap of NK cell for the top 10 DEGs of each NK cell cluster. **(f)** The percentage change tendency and contribution of each NK cell cluster in different groups.

We also explored other clusters of the immune cells in this preliminary study. A total of 1198 B cells were grouped into four clusters. Different groups are shown in the tSNE plot, and the heatmap shows the top 10 DEGs of each B cell cluster (Figure 6a). Although there was only one patient in the SJ group, B cell(0) was the dominant cluster in the SJ group (75%). The CLF group mainly consisted of B cell(0) (53%) and B cell(1) (38%), and the HC group was mostly composed of B cell(2) (50%) and B cell(3) (37%) (Figure 6b). As reported by MacParland *et al*.^24^, B cell(2) appeared to be plasma cells, characterized by the expression of immunoglobulin heavy and light chains. A total of 952 neutrophils were also divided into four clusters, as shown in the tSNE plot. The heatmap shows the top 10 DEGs of each neutrophil cluster (Figure 6c). Neutrophil(0) was the dominant cluster in the SJ group (82%), and neutrophil(0) and neutrophil(1) were the main cluster in the CLF group (35% and 63%, respectively). Neutrophil(0), as well as neutrophil(2) and neutrophil(3), were the dominant clusters in the HC group (23%, 47% and 29%, respectively; Figure 6d). The 2231 NK cells were separated into five clusters, and different groups were shown in the tSNE plot. The heatmap shows the top 10 DEGs of each NK cell cluster (Figure 6e). NK(0) and NK(2) were the predominant clusters in the SJ group(59%, 33%). The CLF group was mainly composed of NK(0) (29%), NK(1) (29%) and NK(2) (28%), while the HC group was mostly composed of NK(3) (61%) (Figure 6f).

## DISCUSSION

Schistosomiasis can still pose a huge economic burden to endemic regions.^25^ Further studies are required to gain understanding of the immune mechanisms associated with hepatic fibrosis in schistosomiasis patients, as the condition remains incompletely defined. Our study employed scRNA‐seq to explore the mechanisms of schistosome‐associated hepatic fibrosis. In this study, we present for the first time a transcriptional atlas of immune cells in the human livers with *Schistosoma japonicum*‐associated fibrosis. Our research also reflects the heterogeneity of liver‐infiltrating immune cells among members of the SJ, CLF and HC groups. This allowed for the identification of immune cell subsets and responding marker genes, as well as the analysis of activated signaling pathways and the functional states of immune cells within the liver. We demonstrated that the upregulated signaling pathway associated with NK cell‐mediated cytotoxicity, and antigen processing and presentation, is related to the mechanism of schistosoma‐associated liver fibrosis. Thus, this study expands our understanding of immune responses in schistosoma‐associated liver fibrosis. Furthermore, our data will provide a reference map of the liver immune landscapes of patients with schistosoma‐associated liver fibrosis.

We collected liver tissues from a cirrhotic patient infected with *S. japonicum* and HBV (the SJ group). Patients with hepatitis B cirrhosis and healthy livers served as controls for the SJ group. After isolating single cells, sequencing was performed on the Illumina Hiseq X Ten sequencing platform. Ultimately, 20 093 cells were included in the data analysis. We found 17 major cell clusters, based on cell‐specific markers. Compared with scRNA‐seq study by MacParland *et al*., we found that the major clusters were MP, T cells, B cells, NK cells, endothelial cells, cholangiocytes and hepatic stellate cells in the healthy control group in both studies. Additionally, we observed that lymphocytes comprise 30% of the non‐parenchymal cells in the liver in the HC group in our study, comparable to the data (about 25%) reported by Racanelli *et al*.^26^

T cells were a major component of the immune cells in the SJ group. T cell(1), MP‐1, DC and endothelial cell‐1 percentages were higher in the SJ group than in the CLF and HC groups, indicating that these cells may play a role in hepatic fibrosis. We performed further analysis of different clusters, finding obvious heterogeneity of different cell types among the different groups. *CAVIN2* ^+^ MP(2) was the predominant MP subset in the SJ group (53%), and it was less dominant in the CLF (8%) and HC (1%) groups. Moreover, Kupffer cell marker genes (*CD163*, *MARCO* and *TIMD4*) were enriched in CAVIN2^+^ MP(2), which was also an important source of *TGFB1*, a precursor of the latency‐associated peptide and transforming growth factor beta‐1 (TGF‐β‐1) chains. The latter of these consists of both the regulatory and active subunits of TGF‐β‐1.^27^ These results imply that *CAVIN2* ^+^ Kupffer cells might strongly affect hepatic fibrosis of schistosomiasis, and that Kupffer cells might promote self‐renewal or differentiation from local progenitors.^28^, ^29^ Controversies regarding schistosoma‐associated liver fibrosis state that coinfection of schistosomiasis with chronic HBV is associated with more severe hepatic fibrosis.^30^, ^31^, ^32^, ^33^ Our results indicated that coinfection of schistosomiasis with chronic HBV might result in worse outcomes. *CAVIN2* ^+^ MP(2) cannot be specifically divided into M1 or M2 due to the low expression of marker genes of M1(*TNF*, *IL1B*, *IL12B*) and M2(*ARG1*, *IL10*). MP(1) with high expression of *LYZ* and *CSTA* in our study was correlated with inflammatory macrophages as reported by MacParland *et al*.,^24^ which also suggested that MP(7) in our study have a tolerogenic function with high expression of *CD5L*, *MARCO*, *VSIG4* and *HMOX1*.^24^ MP(2) KEGG pathways were related to lysosome, endocytosis, phagosome and antigen processing and presentation. T cell(1) and T cell(3) were the major T cells of the SJ group (50% and 32%, respectively) and were an important source of *GZMB*. T cell(1) KEGG pathways were primarily related to NK cell‐mediated cytotoxicity. DC(1) was higher in the SJ group (57%) than in the CLF (16%) and HC (6%) groups. The KEGG pathway of DC(1) was related to Fc gamma R‐mediated phagocytosis and antigen processing and presentation. Our results indicated that most T cells may be able to exert killing effects, and that many DCs might be involved in antigen presentation after maturation of schistosomes in the liver.

Our study has several limitations. A patient infected with *S. japonicum* and HBV was included in the SJ group, and the small number of patients in the SJ group limited our scope. Coinfection of HBV and schistosomiasis was common in the clinic, and we also experienced difficulties when it came to obtaining sufficient liver tissue because of the limited number of schistosomiasis patients receiving liver transplantations.^34^ We included the CLF (*n* = 3) and HC (*n* = 2) groups in our study to compensate for these limitations. In conclusion, we first applied scRNA‐seq to examine schistosome‐associated liver fibrosis and profiled the landscape of immune cells. Furthermore, we will explore the underlying mechanisms of Kupffer cells, T cells and DCs in patients with schistosome‐associated liver fibrosis.

## METHODS

### Human subjects

Human liver tissues were obtained from the Transplantation Center of the 3rd Xiangya Hospital, Central South University. To explore the effects of *S. japonicum* on liver fibrosis, three groups (SJ group, CLF group and HC group) of samples were included. The SJ group (*n* = 1) comprised a single sample of cirrhosis involving infection with *S. japonicum* and HBV. He had an HBV infection history of about 20 years. This patient was clinically diagnosed as having schistosomiasis 40 years ago and had twice received praziquantel chemotherapy. The infection with *S. japonicum* was diagnosed because the patient had a history of exposure to contaminated water, typical clinical manifestations and a positive result for anti‐schistosoma antibodies‐IgG. He was diagnosed as having advanced schistosomiasis in 2007, because of ascites, splenomegaly and cirrhosis. In 2018, this patient received liver transplantation for liver cirrhosis. The CLF group (*n* = 3) comprised hepatitis B cirrhosis. All the patients ultimately received liver transplantation. The two samples in the HC group were obtained from donation after the death of the donors. The characteristics of the enrolled patients are shown in Table 1. Non‐parenchymal cells were collected from the liver for scRNA‐seq. Our study was approved by the Ethics Committee of the 3rd Xiangya Hospital of Central South University and received written informed consent.

**Table 1:** The characteristics of patients

Sample	Gender	Age (year)	ALT (U L^−1^)	AST (U L^−1^)	ALB (g L^−1^)	TB (µmol L^−1^)	DB (µmol L^−1^)	INR	Cre (µmol L^−1^)	Ascites (mL)	Anti‐SJ IgG	HBsAg	MELD
SJ	Male	55	16	31	30.6	35	16.9	1.35	60	2000	Positive	Positive	12
CLF‐1	Male	54	73	63	40.3	27.4	13.9	1.07	165	10000	Negative	Positive	15
CLF‐2	Male	46	28	32	41.1	52.5	16.7	1.37	74	2000	Negative	Positive	14
CLF‐3	Male	37	59	59	30.9	41.7	19.3	1.40	39	1000	Negative	Positive	14
HC‐1	Male	44	29	30	29.9	11.2	3.2	0.99	49	0	Negative	Negative	5
HC‐2	Male	54	29	64	30.8	6.1	2.2	1.02	75	0	Negative	Negative	3

ALB, albumin; ALT, alanine aminotransferase; AST, aspartate aminotransferase; Cre, creatinine; DB, direct bilirubin; INR, international normalized ratio; MELD, model for end‐stage liver disease; TB, total bilirubin.

### Dissociation of the liver into single cells

After surgical resection, the fresh tissues were transported on ice to the Singleron Biotechnologies laboratory in Nanjing, China, as soon as possible. The specimens were washed with Hanks balanced salt solution three times and were minced into 1–2 mm pieces. The tissue pieces were digested at 37°C for 15 min by 2 mL GEXSCOPETM tissue dissociation solution (Singleron Biotechnologies, Nanjing, China). After digestion, the samples were filtered through 40 µm sterile strainers and centrifuged at 320 *g* for 5 min. The supernatant was discarded and 2 mL GEXSCOPETM red blood cell lysis buffer (Singleron Biotechnologies) was added at 25°C for 10 min to remove the red blood cells. After being washed with PBS, the cell suspension was centrifuged at 50 *g* for 5 min to remove hepatocytes from non‐parenchyma cells.

### scRNA‐seq

Single‐cell suspensions with 1 × 10^5^ cells mL^‐1^ were loaded onto microfluidic devices and scRNA‐seq libraries were constructed according to the GEXSCOPETM protocol using the GEXSCOPETM single‐cell RNA library kit (Singleron Biotechnologies). Individual libraries were diluted to 4 nm and pooled for sequencing. Pools were sequenced on a HiSeq X device (Illumina, San Diego, CA, USA) with 150 bp paired end reads.

### scRNA‐seq quantifications and statistical analysis

The Seurat program (http://satijalab.org/seurat/, R package, v.3.2.1) was used to analyze the RNA‐seq data. Unique molecular identifier (UMI) count tables were loaded into R (R version 4.0.2) using the read.table function. KEGG functional enrichment analysis was performed on the gene set using R version 4.0.2 to reveal pathways that were significantly associated with the genes that were specifically expressed.

## CONFLICT OF INTEREST

The authors declare no conflict of interest.

## AUTHOR CONTRIBUTIONS

**Yu Zhang:** Conceptualization; Data curation; Formal analysis; Methodology; Project administration; Software; Writing‐original draft; Writing‐review & editing. **Junhui Li:** Conceptualization; Data curation; Formal analysis; Investigation; Methodology; Project administration; Software; Supervision; Visualization; Writing‐original draft; Writing‐review & editing. **Hao Li:** Data curation; Methodology; Project administration; Software. **Zhaoqin Zhou:** Formal analysis; Investigation; Methodology; Resources. **Chen Guo:** Data curation; Funding acquisition; Investigation; Software. **Jie Jiang:** Data curation; Methodology; Resources. **Yingzi Ming:** Conceptualization; Data curation; Formal analysis; Funding acquisition; Investigation; Methodology; Project administration; Resources; Supervision; Validation; Visualization; Writing‐review & editing.

## Supporting information

Supplementary figure 1
