# Supplementary material for: A preliminary investigation into the immune cell landscape of schistosome‐associated liver fibrosis in humans
Source: Immunol Cell Biol. 2021 Aug 6;99(8):803–13. doi: 10.1111/imcb.12490 (PMC8456952; doi:10.1111/imcb.12490)
Supplement: Supplementary file 1 — Supplementary figure 1 [file IMCB-99-803-s001.pdf]

Supplementary material

Supplementary figure 1

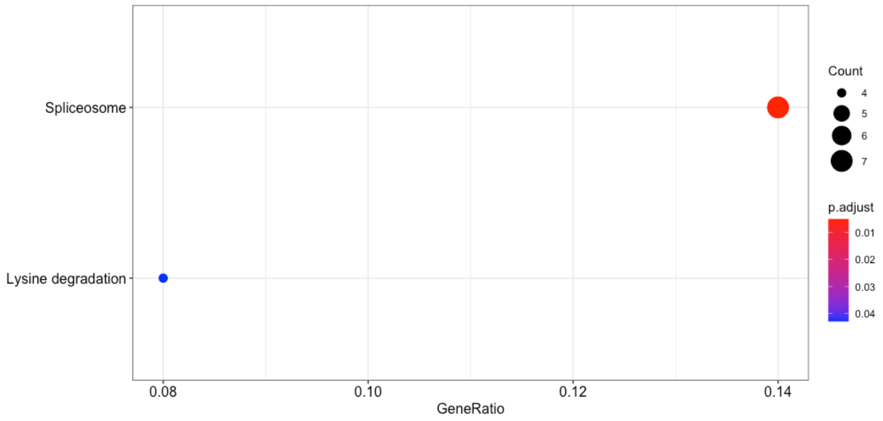

Bubble plots of KEGG pathway enrichment data of T cell(3) vs other T cell.
